# Differential effects of *WRAP53* transcript variants on non-small cell lung cancer cell behaviors

**DOI:** 10.1371/journal.pone.0281132

**Published:** 2023-01-27

**Authors:** Yan Zhu, Wenjie Sun, Xueping Jiang, Rui Bai, Yuan Luo, Yanping Gao, Shuying Li, Zhengrong Huang, Yan Gong, Conghua Xie

**Affiliations:** 1 Department of Radiation and Medical Oncology, Zhongnan Hospital of Wuhan University, Wuhan, Hubei, China; 2 Department of Oncology, The First Affiliated Hospital of Yangtze University, Jingzhou, Hubei, China; 3 Department of Biological Repositories, Zhongnan Hospital of Wuhan University, Wuhan, Hubei, China; 4 Hubei Key Laboratory of Tumour Biological Behaviors, Zhongnan Hospital of Wuhan University, Wuhan, Hubei, China; 5 Hubei Cancer Clinical Study Center, Zhongnan Hospital of Wuhan University, Wuhan, Hubei, China; Duke University School of Medicine, UNITED STATES

## Abstract

**Background:**

The WD40-encoding RNA antisense to p53 (*WRAP53*) is an antisense gene of *TP53* with three transcriptional start sites producing three transcript variants involved in the progression of non-small cell lung cancer. However, the mechanism by which these different transcript variants regulate non-small cell lung cancer cell behaviors is to be elucidated.

**Methods:**

Two non-small cell lung cancer cell lines, A549 cells with wild-type p53 and H1975 with mutated p53, were transfected with *WRAP53*-*1α* and *WRAP53*-*1β* siRNA. The biological effects were assessed via colony formation, cell viability, apoptosis, cell cycle, wound healing and cell invasion assays, as well as immunoblotting.

**Results:**

Knockdown of *WRAP53-1α* increased the mRNA and protein levels of p53; suppressed colony formation and proliferation of A549 cells but promoted them in H1975 cells; increased the proportion of cells in the G0/G1 phase in A549 cells but decreased that in H1975 cells; and suppressed migration and invasion in A549 cells but not in H1975 cells. Conversely, knockdown of *WRAP53-1β* had no effect on p53 expression; promoted the growth of A549 cells but not of H1975 cells; decreased the proportion of cells in the G0/G1 phase in A549 cells but not in H1975 cells; and promoted migration and invasion in A549 cells but not in H1975 cells. Knockdown of both *WRAP53-1α* and *WRAP53-1β* promoted apoptosis in A549 cells but not in H1975 cells.

**Conclusions:**

*WRAP53* transcript variants exerted different functions in non-small cell lung cancer cells and regulated non-small cell lung cancer cell behaviors depending on the p53 expression.

## Introduction

Alternative splicing is a crucial process in gene regulation as it enables a single gene to encode different protein products. It occurs in approximately 95% of multi-exon genes in eukaryotes and is involved in various disorders including cancers [1, 2]. The different transcript variants of a single gene usually have different and even opposite biological functions in physiological and pathological processes.

Mutations in the tumor suppressor p53 (*TP53*) gene not only cause loss of its tumor suppressor function but can also promote an oncogenic function that promotes malignant transformation. Such mutations are found in over 50% of human cancers [3]. *TP53* is the most commonly mutated gene in non-small cell lung cancer (NSCLC) [4]. Mutant p53 plays an important role in the occurrence and progression of NSCLC [5]. Further, *TP53* mutation is a negative prognostic factor in advanced NSCLC and that different mutations have different prognostic values [6]. High expression of mutated p53 is correlated with poor survival, regardless of epidermal growth factor receptor mutation [7].

The *TP53* 5’-untranslated region flanking gene, WD40-encoding RNA antisense to p53 (*WRAP53*), which is located on chromosome 17p13, has three different transcriptional start sites that yield three different variants: 1α, 1β, and 1γ. The start site for the 1α variant corresponds to the first exon of p53 in a cis-antisense manner. *WRAP53*-*1α* is an antisense transcript that stabilizes *TP53* [8]. The *WRAP53*-*1β* transcript is not complementary to *TP53* but encodes the WD repeat-containing protein WRAP53β (WD repeat domain 79) and telomerase Cajal body protein 1, which is involved in multiple cellular processes. WRAP53β regulates the maintenance of the nuclear organelles known as Cajal bodies by recruiting motor neuron proteins, small Cajal body-specific RNAs, and telomerases [9–11]. WRAP53β also targets telomerase to telomeres, promoting their elongation [9]. In addition, WRAP53β facilitates DNA damage repair by recruiting the ubiquitin ligase ring finger protein 8 to DNA breaks during both homologous recombination and non-homologous end joining. The effects of WRAP53β on DNA damage repair are p53-independent, as it also functions in p53-deficient cells [12, 13]. The *WRAP53*-*1γ* transcript is located in the first intron of p53, though its function remains unclear [8].

Moreover, the effects of WRAP53 on cancer progression are controversial. *WRAP53* has been associated with cancer pathogenesis because *WRAP53*-*1α* regulates *TP53* expression. Loss of WRAP53β impairs telomere maintenance and DNA repair, thus increasing genomic instability and the probability of carcinogenesis. It is conceivable that *WRAP53*-*1α and WRAP53-1β* could play different roles in NSCLC cells. To test this hypothesis, we examined the distinct biological function of the two *WRAP53* variants in vitro in cell function assays.

## Materials and methods

### Cell culture

NSCLC cell lines (A549 and NCI-H1975) were purchased from the Type Culture Collection of the Chinese Academy of Sciences (Shanghai, China). Both cell lines were cultured in RPMI-1640 medium (Hyclone, USA) supplemented with 10% fetal bovine serum (Hyclone), 100 U/mL penicillin, and 100 mg/mL streptomycin (Sangon, China). All cells were maintained in a humidified incubator (Sanyo, Japan) at 37°C and 5% CO_2_.

### RNA extraction and quantitative real-time polymerase chain reaction

Total RNA was isolated from cells using TRIzol (Vazyme, China). The RNA concentrations and A260/A280 ratios were assessed with a Nanodrop spectrophotometer (Thermo Scientific, USA). Total RNA (500 ng) was reverse-transcribed using the HiScript® Q RT SuperMix for qPCR kit (Vazyme) according to the manufacturer’s instructions. Quantitative real-time polymerase chain reaction (qRT-PCR) was performed using the ChamQ TM SYBR ® qPCR Master Mix (Vazyme) on the CFX Connect^TM^ RealTime PCR Detection System (Bio-Rad, USA). Glyceraldehyde-3-phosphate dehydrogenase was used as the reference for normalization. The relative fold change of mRNAs was calculated using the 2 ^−ΔΔCt^ method. The primers used for qRT-PCR are presented in Table 1.

**Table 1:** Primer sequences and siRNA sequences used in this study.

Gene	Sequences or target sequence (5’→3’)
GAPDH-F	GGAGCGAGATCCCTCCAAAAT
GAPDH-R	GGCTGTTGTCATACTTCTCATGG
Wrap53-1α-F	CCGGAGCCCAGCAGCTACCTG
Wrap53-1α-R	CATGGCGACTGTCCAGCTTTG
Wrap53-1β-F	AGGAGGGAAGCACAGTATGAAGAC
Wrap53-1β-R	GGCATCAGTTCAGAGTCCGCA
P53-F	ACGACGGTGACACGCTTCCC
P53-R	AGGGGGCTCGACGCTAGGAT
siControl sense	UUCUCCGAACGUGUCACGUTT
siControl Antisense	ACGUGACACGUUCGGAGAATT
siWrap53-1α sense	AAAACCCCAATCCCATCAACC
siWrap53-1α Antisense	AAGGTTGATGGGATTGGGGTT
siWrap53-1β sense	AATCGGAAGGTGGACCGAAAT
siWrap53-1β Antisense	AAATTTCGGTCCACCTTCCGA

### RNA interference

Small interfering RNAs (siRNAs) targeting *WRAP53*-*1α* and *WRAP53*-*1β* [8] and a negative control (NC) siRNAs were purchased from Genepharm Technologies (China). Transient transfection was performed using jetPRIME transfection reagent (Polyplus-transfection® SA, France) following the manufacturer’s recommendations. Briefly, 2.5–3 × 10^5^ cells were seeded in each well of a 6-well plate. After growing the cells to 30–50% confluence, the cells were transfected with NC or target siRNAs.

### Immunoblotting

Cells were lysed in RIPA cell lysis buffer (Beyotime, China) supplemented with phosphatase inhibitors and protease inhibitors (Sigma, USA). The lysates were then centrifuged at 13300 rpm for 15 min at 4°C. The supernatants were collected, and protein concentration was measured using a bicinchoninic acid kit (Beyotime). Equal amounts of protein were applied to a 10–12% sodium dodecyl sulphate-polyacrylamide separating gel and transferred to a polyvinylidene fluoride membrane. The membrane was blocked with 5% non-fat milk and then incubated with primary antibodies at 4°C overnight. After washing, the membrane was incubated with horseradish peroxidase-conjugated secondary antibodies at room temperature for 1 h. An enhanced chemiluminescence kit (Bio-Rad) was used to detect the proteins under a gel imaging analyzer (Bio-Rad). Antibodies used for immunoblotting are listed in Table 2.

**Table 2:** Antibodies used in this study.

Antibody	Company	Dilution
GAPDH (10494-1-AP)	Proteintech	1:2500
WRAP53 (14761-1-AP)	Proteintech	1:1000
P53 (10442-1-AP)	Proteintech	1:1000
E-Cadherin (20874-1-AP)	Proteintech	1:1000
Vimentin (10366-1-AP)	Proteintech	1:1000
MMP9 (10375-2-AP)	Proteintech	1:1000
BAX (60267-1-lg)	Proteintech	1:5000
BCL-2 (60178-1-lg)	Proteintech	1:1000
CDK2 (10122-1-AP)	Proteintech	1:1000
CDK4 (11026-1-AP)	Proteintech	1:1000
CyclinE1 (11554-1-AP)	Proteintech	1:1000
CyclinD1 (60186-1-lg)	Proteintech	1:5000
P21 (10355-1-AP)	Proteintech	1:1000
Tubulin (10094-1-AP)	Proteintech	1:1000

### Colony formation assay

After transfection, tumor cells were plated in 6-well plates (500 cells/well) and cultured for 2 weeks. Cell colonies were fixed with 4% paraformaldehyde (Sangon) for 20 min and stained with 0.5% crystal violet (Beyotime) at room temperature for 30 min. After washing the wells with water, the number of colonies in each well was counted under a microscope. This assay was repeated three times and performed in triplicates.

### Cell viability assay

Cell counting kit-8 (CCK-8) was used to quantify cell viability. The CCK-8 method is based on the conversion of the water-soluble tetrazolium WST-8 to a water-soluble formazan dye upon reduction by dehydrogenases in the presence of an electron carrier. All the experiments were conducted according to the manufacturer’s protocol. Transfected cells were seeded into 96-well plates (3000 cells/well in 100 μL medium). After culturing for 24, 48, 72, and 96 h, the supernatant was replaced with 100 μL serum-free medium with 10 μL CCK-8 (Dojindo, Japan) and incubated at 37°C in the dark for 1 h. The absorbance was measured at 450 nm by a microplate reader (Rayto, China). Each group had 5 wells, and the experiment was conducted three times.

### Apoptosis assay

Cells were seeded into 6-well plates and transfected with NC or *WRAP53* siRNAs. The cells were harvested 48 h later, washed twice with cold phosphate-buffered saline (PBS), and stained with fluorescein isothiocyanate-conjugated annexin V (BioLegend, China) for 20 min and propidium iodide (BD, USA) in the dark for 5 min. The stained cells were assessed by flow cytometry (Beckman, China) and analyzed by CytExpert 2.3 software (Beckman). Each experiment was conducted three times.

### Cell cycle analysis

Cells were harvested with trypsin without EDTA (Corning, USA) 48 h after transfection. After washing with cold PBS, the cells were treated with a cell cycle staining kit (MultiSciences Biotech, China). Flow cytometry (Beckman) was used to detect the percentage of cells in different cell cycle phases. This assay was repeated at least three times.

### Wound healing assay

Cells were seeded in 6-well plates and transfected with NC or *WRAP53* siRNAs. When the cells were confluent, they were scratched with a 10-μL pipette tip and washed with PBS gently to remove cell debris. After that, 2 mL serum-free medium was added into each well, and pictures were taken using a phase contrast microscope (Nikon Microphot-FX, Japan) at 0, 24, and 48 h. The migration rate was calculated as follows:

Wound closure (%) = (distance of initial scratch−distance of closed scratch)/distance of initial scratch.

### Cell invasion assays

Cells (1 × 10^5^ cells/well) transfected with NC or *WRAP53* siRNAs were seeded in the upper chambers of transwell units pre-coated with 100 μL Matrigel (BD, USA, dilution 1:40). After incubation for 24 h, the filters were fixed with 4% paraformaldehyde for 20 min and stained with 0.5% crystal violet for 20 min. The cells on the upper surface of the filter were completely removed using a cotton swab. The cells that invaded through the Matrigel and reached the lower surface of the filter were counted. Experiments were independently repeated for three times.

### Statistical analysis

All statistical analyses were performed with GraphPad version 5.0 software (GraphPad, USA). All data are presented as the mean ± standard deviation (SD). Unpaired two-tailed student’s t-test was used for comparisons between groups. A P value < 0.05 was considered statistically significant.

## Results

### Knockdown of *WRAP53*-*1α*, but not *WRAP53-1β*, induced p53 expression

**Fig 1:**
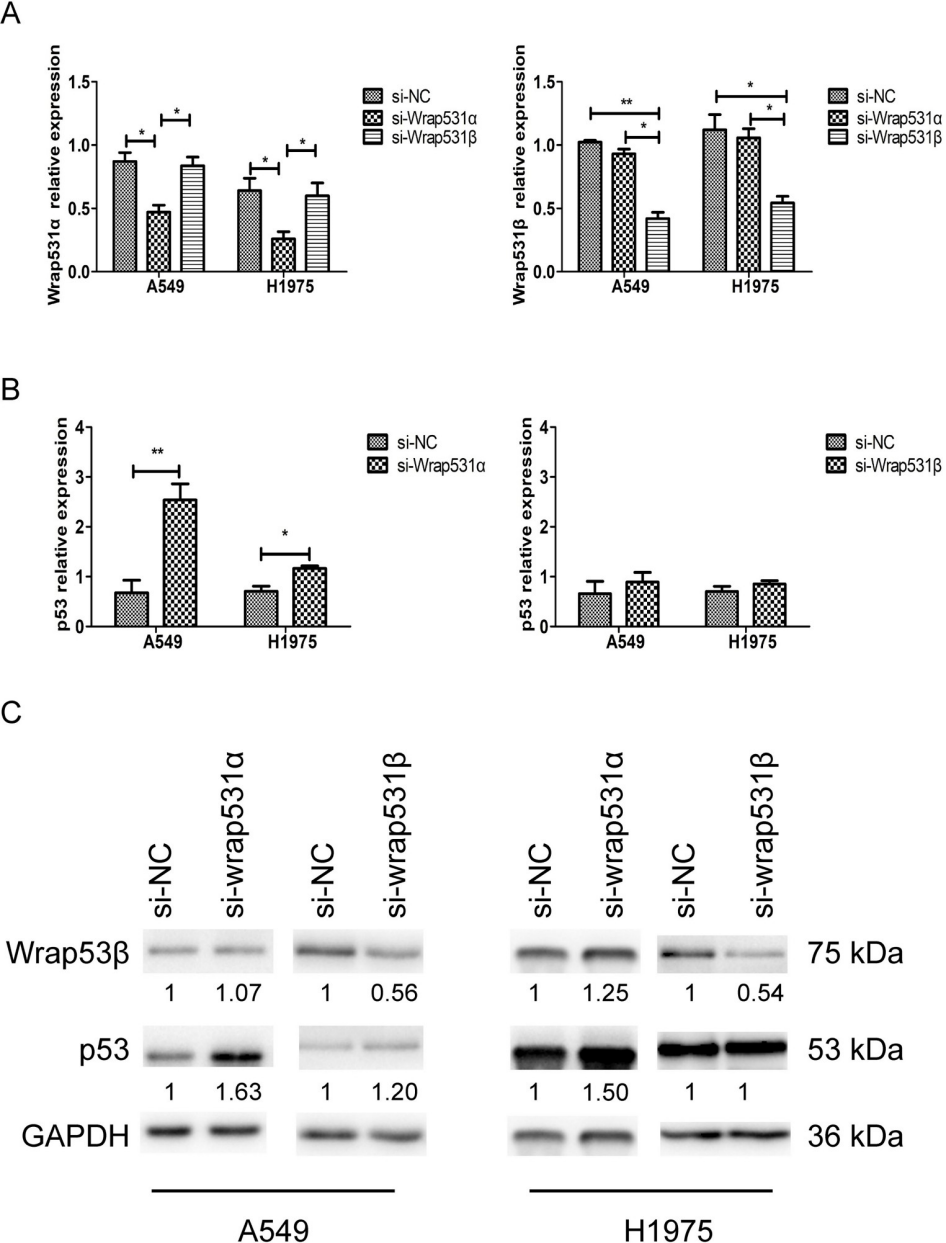
Knockdown of *WRAP53*-1*α* increases p53 expression. (A) qRT-PCR analysis of *WRAP53*-*1α* and *WRAP53*-*1β* mRNA levels in A549 and H1975 cells transfected with negative control siRNA (si-NC), *WRAP53*-*1α* siRNA (si-WRAP53-1α), or *WRAP53-1β* siRNA (si-WRAP53-1β). (B) qRT-PCR analysis of *TP53* mRNA levels in A549 and H1975 cells transfected with si-NC, si-WRAP53-1α, or si-WRAP53-1β. (C) Immunoblotting of WRAP53-1β and p53 in A549 and H1975 cells transfected with si-NC, si-WRAP53-1α, or si-WRAP53-1β. GAPDH was used as an internal control. N = 3; *, P < 0.05; **, P < 0.01.

Transcript-specific siRNAs were designed to specifically target *WRAP53*-*1α* and *WRAP53*-*1β*. There was no marked difference in *WRAP53*-*1α* mRNA levels between cells transfected with si-Control and si-WRAP53-1β, whereas there was significantly less *WRAP53*-*1α* mRNA in A549 and H1975 cells transfected with si-WRAP53-1α (P < 0.05; Fig 1A). Similarly, knockdown of *WRAP53-1β* reduced *WRAP53*-*1β* RNA levels in A549 and H1975 cells (P < 0.01 and P < 0.05, respectively; Fig 1A). qRT-PCR analysis showed that knockdown of *WRAP53*-*1α* significantly increased *TP53* mRNA levels in both A549 and H1975 cells, whereas knockdown of *WRAP53-1β* had no effect on *TP53* levels (Fig 1B). Immunoblotting analysis showed that there was no difference in WRAP53β protein levels between cells transfected with si-Control and si-WRAP53-1α, whereas the expression of WRAP53β protein was decreased in cells transfected with si-WRAP53-1β. Additionally, knockdown of *WRAP53*-*1α* increased p53 protein levels in both A549 and H1975 cells, whereas knockdown of *WRAP53-1β* had no effect (Fig 1C).

### *WRAP53*-*1α* and *WRAP53-1β* had different effects on NSCLC cell growth and proliferation

**Fig 2:**
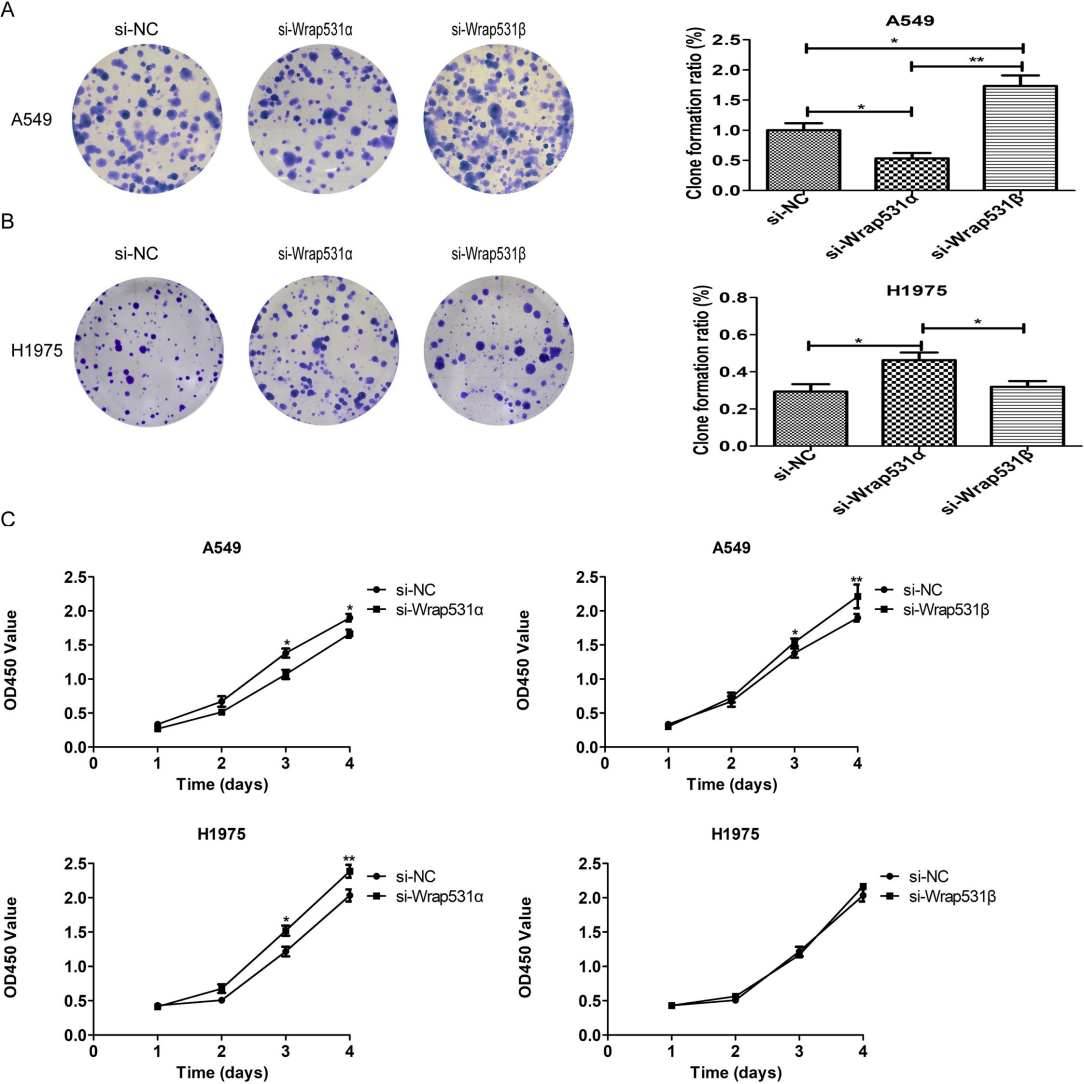
*WRAP53*-*1α* and WRAP53-1β have different effects on NSCLC cell proliferation. (A) Representative images of colony formation assays using A549 cells transfected with *WRAP53* or control siRNA. Knockdown of *WRAP53*-1*α* reduced colony formation, whereas knockdown of *WRAP53-1β* had the opposite effects. (B) Knockdown of *WRAP53*-1*α* increased colony formation in H1975 cells. (C) Proliferation of cells transfected with si-WRAP53-1α or si-WRAP53-1β was measured by CCK-8 assay. Knockdown of *WRAP53*-*1α* reduced A549 cell proliferation, whereas knockdown of *WRAP53-1β* increased proliferation. In H1975 cells, depletion of *WRAP53*-*1α* increased proliferation, and depletion of *WRAP53-1β* had no effect. N = 3; *, P < 0.05; **, P < 0.01.

To investigate the functions of *WRAP53*-*1α* and *WRAP53-1β* in NSCLC cell growth, both A549 and H1975 cells were transfected with si-WRAP53-1α or si-WRAP53-1β. The colony formation assay revealed that colony formation in A549 cells was significantly decreased after *WRAP53*-*1α* knockdown but significantly increased after *WRAP53-1β* knockdown (Fig 2A). Conversely, colony formation in H1975 cells was promoted by *WRAP53*-*1α* knockdown, and *WRAP53-1β* knockdown had no significant effect (Fig 2B). The cell viability assay revealed that *WRAP53*-1*α* knockdown suppressed A549 cell proliferation, while *WRAP53-1β* knockdown significantly promoted it (Fig 2C). Conversely, *WRAP53*-*1α* knockdown induced H1975 cell proliferation, while *WRAP53-1β* knockdown had no significant effect (Fig 2C). These results suggested that *WRAP53*-*1α* and *WRAP53-1β* have distinct effects on NSCLC cell proliferation and function depending on the p53 mutation.

### *WRAP53*-*1α* and *WRAP53-1β* deficiency induced apoptosis in NSCLC cells

**Fig 3:**
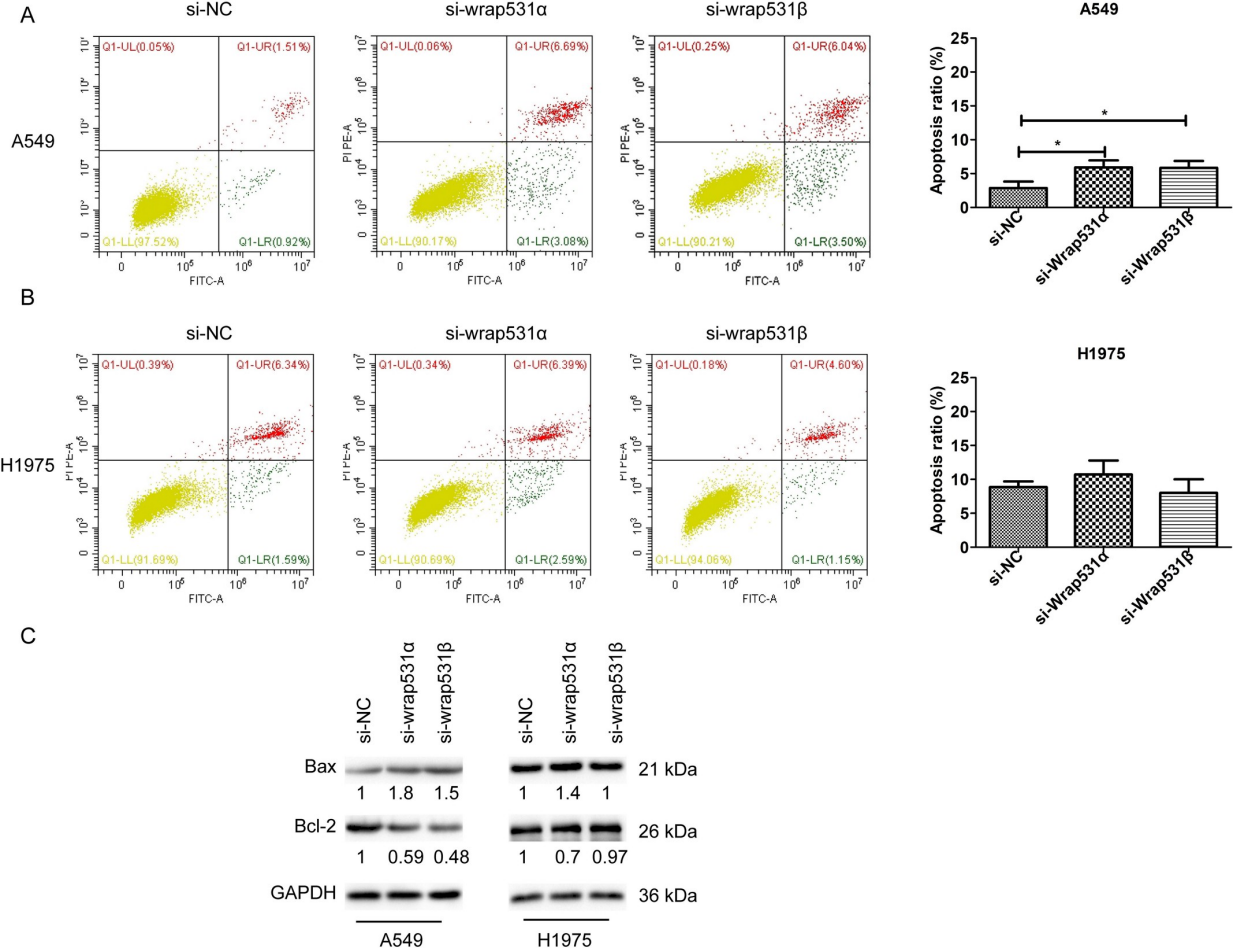
Knockdown of *WRAP53*-*1α* and *WRAP53-1β* promotes apoptosis in A549 cells but not in H1975 cells. (A) Knockdown of *WRAP53*-*1α* and *WRAP53-1β* increased apoptosis in A549 cells. N = 3; *, P < 0.05. (B) Flow cytometry indicated that there was no significant difference in apoptosis in H1975 cells transfected with control, *WRAP53*-*1α*, or *WRAP53-1β* siRNA. (C) Protein levels of the apoptosis-related markers, Bax and Bcl-2, were assessed by immunoblotting.

Flow cytometry was used to measure apoptosis in NSCLC cells transfected with si-WRAP53-1α or si-WRAP53-1β. Knockdown of either *WRAP53*-*1α* or *WRAP53-1β* induced apoptosis in A549 cells (P < 0.05; Fig 3A), but neither knockdown had any significant effect on apoptosis in H1975 cells (Fig 3B). Immunoblotting results indicated that knockdown of either *WRAP53*-*1α* or *WRAP53-1β* upregulated B cell lymphoma 2 (Bcl-2) associated X protein and downregulated Bcl-2 in A549 cells. However, these changes were mild in H1975 cells transfected with si-WRAP53-1α or si-WRAP53-1β (Fig 3C). These results suggested that *WRAP53*-*1α* and *WRAP53-1β* negatively regulated apoptosis in cells with wild-type p53 (A549) but not in cells with mutant p53 (H1975).

### *WRAP53*-*1α* and *WRAP53-1β* had different effects on cell cycle progression in NSCLC cells

**Fig 4:**
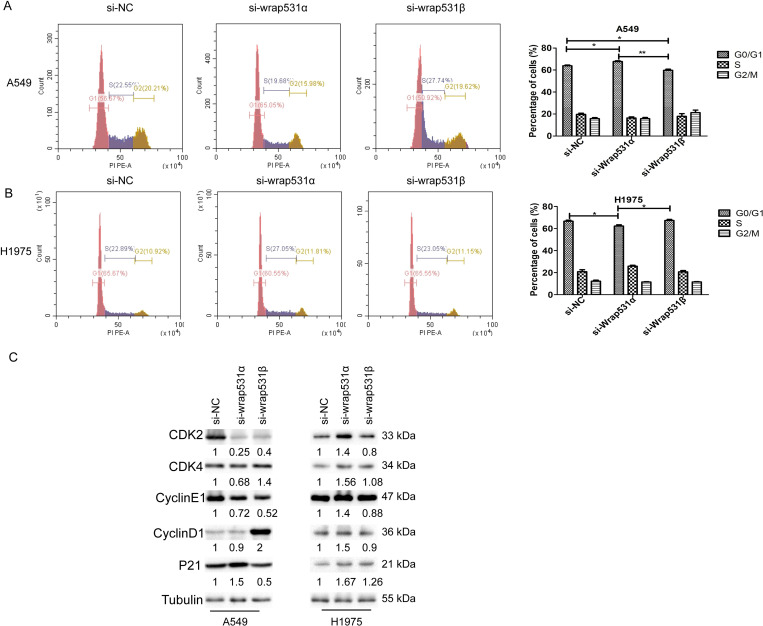
*WRAP53*-*1α* and *WRAP53-1β* have different effects on the cell cycle in NSCLC cells. (A) In A549 cells, knockdown of *WRAP53*-*1α* increased the proportion of cells in the G0/G1 phase, whereas *WRAP53-1β* knockdown decreased the proportion of cells in the G0/G1 phase. (B) In H1975 cells, knockdown of *WRAP53*-*1α* decreased the proportion of cells in the G0/G1 phase. (C) Protein levels of cell cycle regulation and checkpoint proteins were assessed by immunoblotting. N = 3; *, P < 0.05; **, P < 0.01.

Cell cycle distribution was assessed by flow cytometry. In A549 cells, G0/G1 arrest was significantly induced by *WRAP53*-*1α* knockdown, whereas the proportion of cells in the G0/G1 phase decreased by *WRAP53-1β* knockdown (Fig 4A). However, in H1975 cells, the proportion of cells in the G0/G1 phase was decreased by *WRAP53*-*1α* knockdown (P < 0.05); *WRAP53-1β* knockdown had no significant effect on the cell cycle (Fig 4B). To verify the impacts of *WRAP53*-*1α* and *WRAP53-1β* on the cell cycle, the levels of cell cycle regulation and checkpoint proteins were measured by immunoblotting. Cyclin-dependent kinase 4 (CDK4) was downregulated by knockdown of *WRAP53*-*1α* and upregulated by knockdown of *WRAP53-1β* in A549 cells. However, in H1975 cells, the levels of CDK4 were increased by knockdown of *WRAP53*-*1α* but not affected by knockdown of *WRAP53-1β* (Fig 4C). These results suggested that *WRAP53*-*1α* and *WRAP53-1β* had diverse effects on NSCLC cell cycle arrest, and the regulation was p53 mutation-dependent.

### *WRAP53*-*1α* and *WRAP53-1β* had different effects on migration and invasion in NSCLC cells

**Fig 5:**
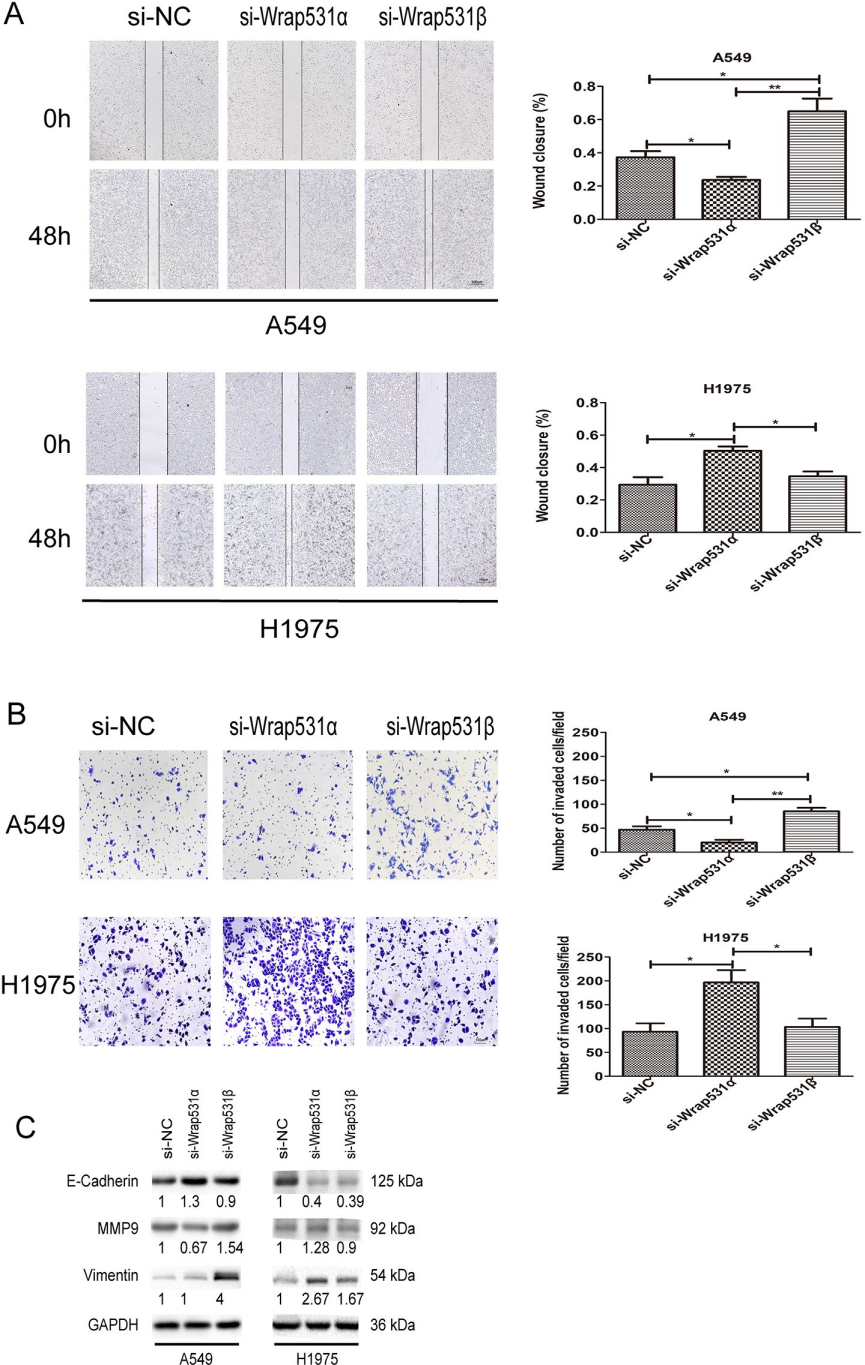
*WRAP53*-*1α* and *WRAP53-1β* have different effects on the invasion and migration of NSCLC cells. (A) Wound healing assays indicated that knockdown of *WRAP53*-*1α* blocked wound closure and that *WRAP53-1β* knockdown promoted wound closure in A549 cells. Knockdown of *WRAP53*-*1α* accelerated wound closure in H1975 cells. (B) Modified Boyden chamber assays indicated that knockdown of *WRAP53*-*1α* reduced cell invasion and that knockdown of *WRAP53-1β* increased cell invasion in A549 cells. Knockdown of *WRAP53*-*1α* increased invasion in H1975 cells. (C) Protein levels of the invasion-related markers E-cadherin, MMP9, and vimentin were assessed by immunoblotting. Scale bar, 100 μm. N = 3; *, P < 0.05; **, P < 0.01.

The impacts of *WRAP53*-*1α* and *WRAP53-1β* on migration and invasion in NSCLC cells were examined by wound healing and modified Boyden chamber assays. A549 cell migration was significantly suppressed by *WRAP53*-*1α* knockdown, but promoted by *WRAP53-1β* knockdown (Fig 5A). In contrast, H1975 cell migration was induced by *WRAP53*-*1α* knockdown (P < 0.05); *WRAP53-1β* knockdown had no significant effect (Fig 5A). According to the results of the modified Boyden chamber assay, A549 cell invasion was suppressed by *WRAP53*-*1α* knockdown, but promoted by *WRAP53-1β* knockdown (Fig 5B). In contrast, H1975 cell invasion was promoted by *WRAP53*-*1α* knockdown (P < 0.05); *WRAP53-1β* knockdown had no significant effect (Fig 5B). Immunoblotting demonstrated that matrix metalloproteinase 9 (MMP9) was downregulated by *WRAP53*-*1α* knockdown and upregulated by *WRAP53-1β* knockdown in A549 cells. However, MMP9 was upregulated by *WRAP53*-*1α* knockdown and not affected by *WRAP53-1β* knockdown in H1975 cells (Fig 5C). These results suggested that *WRAP53*-*1α* and *WRAP53-1β* had distinct effects on migration and invasion in NSCLC cells and functioned depending on the p53 mutation.

## Discussion

In the present study, the two *WRAP53* transcript variants, *WRAP53*-*1α* and *WRAP53*-*1β*, were knocked down in A549 cells (which have wild-type p53) and H1975 cells (which have mutated p53). *WRAP53*-*1α* regulated both the wild-type p53 and mutant p53 expressions. Knockdown of *WRAP53*-*1α* showed anti-tumor effects in A549 cells, but it had the opposite effects on H1975 cells. *WRAP53*-*1α* regulated NSCLC cell behaviors based on p53 mutation. In contrast, *WRAP53-1β* did not regulate the expression of either wild-type or mutant p53 and acted as a tumor suppressor in A549 cells but without effect in H1975 cells. Our results suggest that the two *WRAP53* transcript variants have distinct effects on p53 and NSCLC cells.

Natural antisense transcripts are long non-coding RNAs that occur naturally and play important roles in carcinogenesis, invasion, and metastasis [14]. The long non-coding RNA *WRAP53-1α* is a naturally occurring p53 antisense transcript that acts as a crucial effector in several cancers [15, 16]. *WRAP53-1α* is upregulated by anticancer drugs, and miR-4732-5p has a binding site in the 5’-untranslated region of *WRAP53* [17–19]. In addition, *WRAP53-1α* methylation is significantly associated with worse survival in NSCLC. It is worth noting that *WRAP53-1α* stabilizes *TP53* mRNA to increase the tumor suppressor activity of wild-type p53, leading to better prognosis. However, downregulation of *WRAP53-1α* by promoter methylation does not affect survival in p53-mutated tumors [20]. These results suggest that *WRAP53-1α* regulates p53 signaling. The role of *WRAP53-1α* had not been studied in NSCLC cells.

WRAP53β, a WD repeat-containing protein, also acts as a tumor suppressor to regulate various cellular activities [21]. The significance of WRAP53β in tissue homeostasis is demonstrated by the finding that inherited mutations in WRAP53β lead to telomere dysfunction and dyskeratosis congenita, increasing the risk of tumorigenesis [22]. Moreover, single nucleotide polymorphisms and downregulation of WRAP53β are associated with various sporadic forms of cancer, including breast and ovarian cancer [23–26]. In addition, WRAP53β downregulation is correlated with resistance of head and neck cancer to radiotherapy [27], as well as disruption of the DNA damage response in ovarian tumors [28].

WRAP53β is a potential oncoprotein whose overexpression leads to transformation and promotes cancer cell survival and whose downregulation induces massive cell death [29–32]. Additionally, overexpression of WRAP53β is related to NSCLC progression. Knockdown of WRAP53β significantly inhibits the proliferation of NSCLC cells both *in vitro* and *in vivo* by inducing cell cycle arrest and apoptosis. WRAP53β induces cell cycle arrest at the G0/G1 phase and regulates the expression of G0/G1-related cyclins and cyclin-dependent kinase complexes. WRAP53β knockdown was also reported to induce apoptosis via the mitochondrial pathway [33]. WRAP53β colocalizes and interacts with ubiquitin-specific protease-7, which reduces the ubiquitination of murine double minute 2 and p53, thereby extending the half-life of these proteins and increasing their stability [34]. Additionally, WRAP53β exerts a proliferative effect on NSCLC cells via stabilizing ubiquitin-like containing PHD and RING finger domains 1 [35]. Moreover, WRAP53β may be related to p53 mutations and acts as an independent biomarker to predict poor prognosis of patients with surgically resected NSCLC [36].

The involvement of *WRAP53* in disease progression is evident in lung cancer, but whether different targets of *WRAP53* variants exert different biological functions still requires further investigation. In the present study, we used different variant-specific siRNAs to knock down *WRAP53*-*1α* and *WRAP53-1β* and evaluated the functions of these variants in NSCLC cells. First, *WRAP53*-*1α* was significantly associated with p53 expression, unlike *WRAP53-1β* expression, which is consistent with the results of a previous study [8]. However, knockdown of *WRAP53-1α* upregulated p53 in A549 and H1975 cells, which conflicts with the results of a previous study wherein *WRAP53*-*1α* stabilized p53 [8, 20]. At the molecular level, Natural antisense transcripts can have a concordant or discordant relationship with the sense transcript, and this interaction may result in higher stability or translational repression of the sense RNA [37]. Therefore, we believe that *WRAP53*-*1α* might play different roles based on the p53 mutation status in NSCLC cells.

We further demonstrated that *WRAP53*-*1α* had different effects on NSCLC cell behaviors. It is highly probable that the different mutation statuses of p53 led to these contradictory biological functions. The tumor suppressor p53 is a cellular gatekeeper that guards against genetic abnormality and instability via sensing multiple stress signals, including DNA damage and oncogene activation [38]. In addition, mutations in p53 usually result in increased half-life and nuclear accumulation, which can promote cancer progression via subverting multiple tumor suppression pathways [39]. In A549 cells, knockdown of *WRAP53*-*1α* induced upregulation of wild-type p53 and activation of cell cycle arrest and apoptosis, increased protein levels of cell cycle inhibitor p21 and pro-apoptotic Bax, and decreased the expression of pro-survival Bcl2 in A549 cells. However, in H1975 cells, which harbor a p53 mutation, the knockdown of *WRAP53*-*1α* exerted the opposite effects on cell cycle arrest and had no effect on apoptosis. The p53 has been reported to directly or indirectly regulate the expression of various epithelial mesenchymal transition markers [40]. In the present study *WRAP53*-*1α* knockdown increased the expression of epithelial marker E-cadherin and decreased the expression of mesenchymal markers MMP9 in A549 cells but demonstrated the opposite effects in H1975 cells. These results indicated that *WRAP53*-*1α* regulated cell proliferation, survival, and epithelial mesenchymal transition depending on p53 mutation.

Cell function assays indicated that *WRAP53-1β* acts as a tumor suppressor in A549 cells. Knockdown of *WRAP53-1β* induced slight apoptosis and promoted the G1/S phase transition in A549 cells but had no effect on H1975 cells. The expressions of mesenchymal markers MMP9 and Vimentin were upregulated by the knockdown of *WRAP53-1β* in A549 cells. Though *WRAP53*-*1β* knockdown decreased the expression of E-cadherin and slightly increased the expression of Vimentin, MMP9 was not affected by the knockdown of *WRAP53-1β* in H1975 cells. These findings concerning the contribution of WRAP53-1β to cancer may occur depending on the p53 mutation, which is consistent with the findings in animal models [36]. Inactivation of *WRAP53-1β* could help initiate tumor development by impairing telomere maintenance and DNA repair, leading to genomic instability. Our data suggested that *WRAP53-1β* is a putative tumor suppressor during the progression and metastasis of NSCLC, whereas *WRAP53*-*1α* may have a dual function, thereby suggesting potential interactions between the two transcript variants. This study provides a foundation for further examining the mechanisms by which *WRAP53*-*1α* and *WRAP53-1β* exert their functions in NSCLC, but more *in vivo* validation is needed.

The roles of *WRAP53* isoforms in p53 regulation, telomerase/telomere function, and DNA damage repair are to be investigated, but whether the various molecular functions are independent of each other is still not completely clear. The cDNA microarray showed that *WRAP53* directly or indirectly mediated the factors related to tumorigenesis, such as *p53* signaling pathway, cell cycle and apoptosis pathway, Jak-STAT signaling pathway, PI3K-Akt signaling pathway [30]. We hypothesized that *WRAP53-1α* mainly plays biological functions by regulating p53, while *WRAP53-1β* plays various functions, such as regulating telomerase and DNA damage repair.

## Conclusions

Different transcript variants of *WRAP53* played different and even opposite roles in NSCLC cells. NSCLC therapeutic strategies may include specifically targeting *WRAP53* variants.

## Supporting information

S1 Raw images Complied all original uncropped and unadjusted blot images. (PDF)
