# Supplementary material for: Differential effects of WRAP53 transcript variants on non-small cell lung cancer cell behaviors
Source: PLoS One. 2023 Jan 27;18(1):e0281132. doi: 10.1371/journal.pone.0281132 (PMC9882892; doi:10.1371/journal.pone.0281132)

Fig. 1C original uncropped and unadjusted blot images.

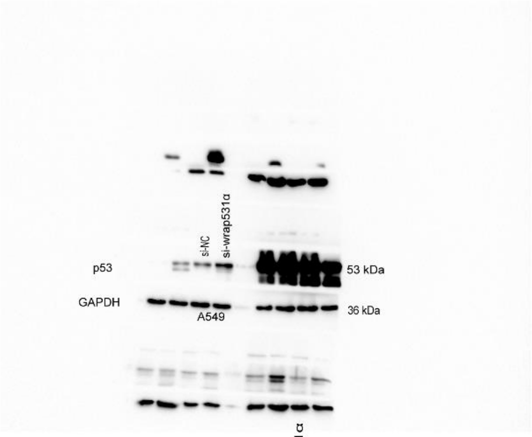

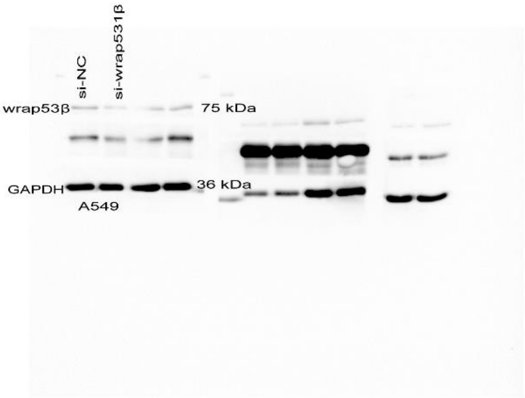

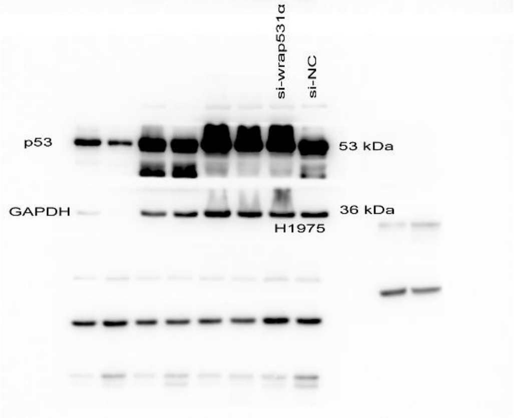

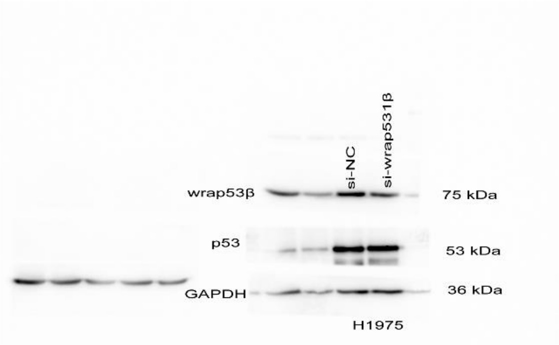

Fig. 3C original uncropped and unadjusted blot images.

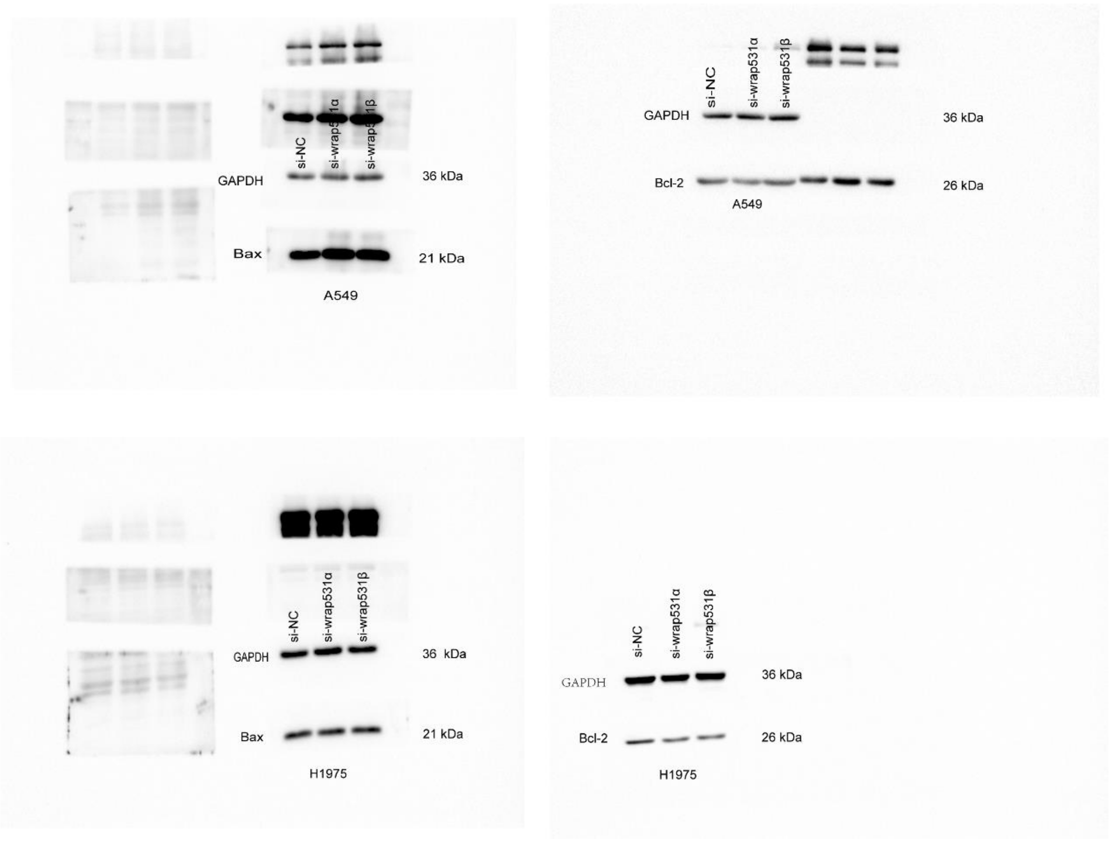

Fig. 4C original uncropped and unadjusted blot images.

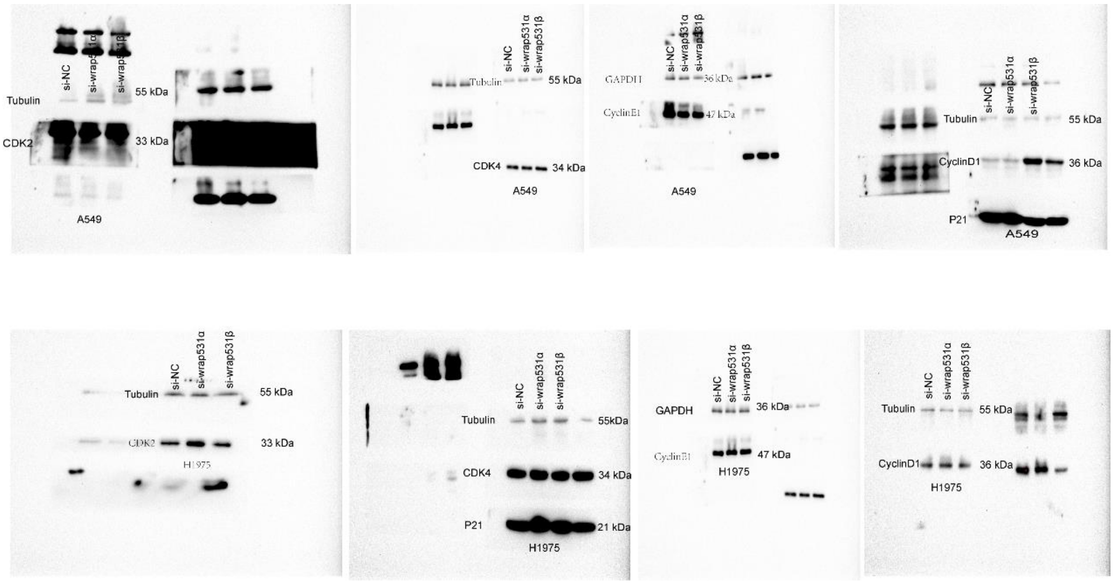

Fig. 5C original uncropped and unadjusted blot images.

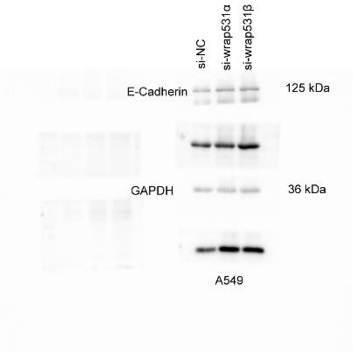

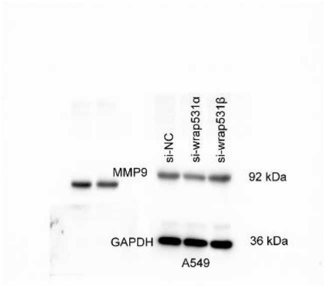

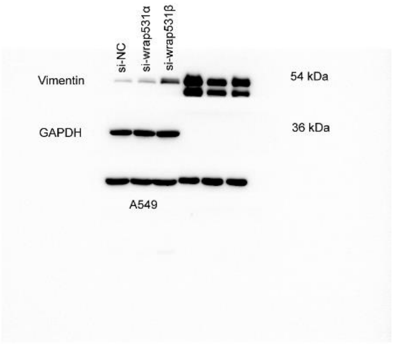

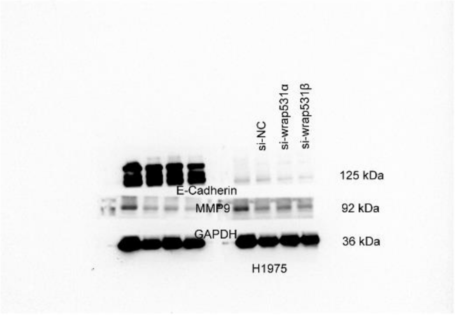

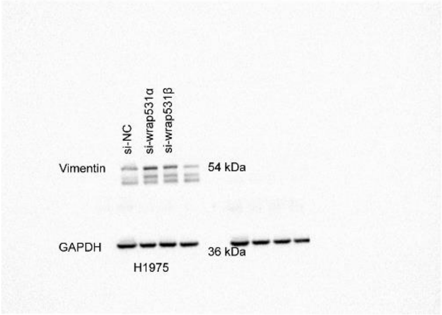

Supplement: S1 Raw images — (PDF) [file pone.0281132.s001.pdf]
